# Clinical Management of a Patient with Chronic Recurrent Vertigo Following a Mild Traumatic Brain Injury

**DOI:** 10.1155/2009/910596

**Published:** 2009-10-08

**Authors:** Eric G. Johnson

**Affiliations:** Department of Physical Therapy, Loma Linda University, Loma Linda, CA 92350, USA

## Abstract

Vertigo, was provoked and right torsional up-beat nystagmus was observed in a 47-year-old patient when she was placed into the right Hallpike-Dix test position using infrared goggle technology. The clinical diagnosis was benign paroxysmal positional vertigo (BPPV), specifically right posterior canalithiasis, resulting from a mild traumatic brain injury (TBI) suffered approximately six-months earlier. Previous medical consultations did not include vestibular system examination, and Meclizine was prescribed to suppress her chief complaint of vertigo. Ultimately, the patient was successfully managed by performing two canalith repositioning maneuvers during a single clinical session. The patient reported 100% resolution of symptoms upon reexamination the following day, and the Hallpike-Dix test was negative. Continued symptom resolution was subjectively reported 10 days postintervention via telephone consultation. This case report supports previous publications concerning the presence of BPPV following TBI and the need for inclusion of vestibular system examination during medical consultation.

## 1. Introduction

Benign paroxysmal positional vertigo (BPPV) is a common vestibular system pathology affecting people of all ages, although the elderly people are most likely to suffer from the problem [1]. BPPV is a condition where the calcium carbonate crystals (otoconia) in the utricle become dislodged and travel through the endolymphatic fluid into one of the three semicircular canals (SCC) of the inner ear [2]. Symptoms consistent with BPPV include brief attacks of vertigo and nausea that are provoked by angular position changes such as bending forward or sitting up in bed [2, 3]. Mechanisms causing BPPV include age-related degeneration of the gelatinous matrix within the utricle supporting the otoconia, infection, and trauma [3]. Symptoms can self-resolve within several days to weeks or may persist chronically [3]. The primary clinical examination used to make the diagnosis of BPPV is the Hallpike-Dix test, and infrared goggles can be used additionally to improve the accuracy of the test. The Hallpike-Dix test involves the examiner taking the patient from a long-sitting position, with their head rotated approximately 45 degrees to one side, into a supine lying with their head and neck extended slightly below the level of the table while maintaining the rotated head position [3]. Due to the gravitational impact of the test position on the SCC's, otoconia within the SCC's begin to move. This increases the internal pull on the endolymphatic fluid of the SCC producing a more vigorous bending of the cilia within the ampulla of the SCC [2, 3]. The end result of the Hallpike-Dix test, in the presence of BPPV, is vertical-torsional geotropic jerk nystagmus of typically short duration suggesting a specific type of BPPV called canalithiasis. Of the three SCC's within each inner ear, the most common canal involved is the posterior SCC due to its anatomical position relative to the utricle [2, 3]. The most effective clinical intervention for canalithiasis is the canalith repositioning maneuver (CRM), also known as the Epley maneuver, which can be performed specific to the individual SCC and side of involvement [2–6].

## 2. Methods

**Case**

A 47-year-old female patient, with a chief complaint of chronic recurrent vertigo since striking her head on the concrete after falling approximately 6 months earlier, was examined for possible BPPV. She received several medical consultations including the initial emergency room visit immediately after the injury. The original course of symptoms included daily nausea, emesis, and vertigo that persisted for about 2 weeks before subsiding. Meclizine was prescribed to manage her symptoms during that time. Several months later, her symptoms spontaneously returned. At the time of the present consultation, she was referred by her primary care physician with a diagnosis of chronic vertigo. The subjective examination revealed intermittent position-provoked vertigo lasting less than 1 minute after gross angular position changes and nausea. Primary provocative position changes were rolling over in bed or bending towards her right side. She denied recent emesis. The physical examination included screening tests for cervical instability and cervical positional tolerance suggestive of vascular patency [7, 8]. Once it was determined that the cervical screening examinations were negative, a left Hallpike-Dix test was performed using infrared goggle technology and it was negative. The patient was returned to the long-sitting position and after a brief rest, a right Hallpike-Dix test was performed. This test produced the patient's vertigo and nausea and multiple beats of robust right torsional up-beat nystagmus, persisting for about 20 seconds before fatiguing, were observed. A CRM was performed and the patient was returned to a sitting position. The patient was agreeable to performance of a subsequent right Hallpike-Dix test within a couple of minutes which again produced her nausea, but nystagmus was not observed. A subsequent CRM was performed and after returning her to a sitting position, she was instructed to avoid sleeping on her right side that night and avoid angular position changes as able until her followup examination scheduled for the following day.

## 3. Results

The patient reported 100% resolution of both her vertigo and nausea during the followup examination the next day. A right Hallpike-Dix was performed and judged to be negative because nystagmus was not observed and there were no reports of vertigo or nausea. The patient was telephoned 10 days later and she continued to report 100% resolution of all symptoms. The patient was discharged at this time.

## 4. Discussion

The mechanism driving BPPV is otoconia pathologically located in one of the three SCC's of the inner ear. While usually idiopathic, otoconia from the utricle can find its way into the posterior SCC after an infection, structural degeneration, or trauma [2, 3]. Because otoconia have mass, angular position changes impact the involved SCC as the otoconia move within the endolymphatic fluid. This movement results in an increased neural firing rate of the involved inner ear and a sensory mismatch between the systems responsible for position-sense and balance [2, 3]. The symptomatic consequence includes nausea and vertigo lasting seconds to minutes [2, 3]. Pharmacological agents such as Meclizine and Phenergan may be prescribed to suppress the vertigo and nausea, respectively. Medical management aimed at resolving BPPV includes the CRM and/or liberatory maneuvers depending upon the specific BPPV type. The purpose of these interventions is to physically remove the otoconia from the SCC and relocate it back into the utricle using gravity and a systematic progression of head position changes [2, 3]. 

In the case presented above, the patient reported chronic recurrent vertigo and nausea resulting from a mild traumatic brain injury suffered in a fall 6 months prior. Despite her complaint of vertigo and several medical consultations, examination of her vestibular system, including the potential for BPPV, was not performed. A growing body of evidence suggests that BPPV is a more common finding than previously thought following head trauma, and there is general consensus that the vestibular system should be included in the examination process [4–6, 9–11]. 

Bertholon et al. [4] reported BPPV in three patients subsequent to head trauma. All three patients responded rapidly to the CRM, and the authors proposed that “early diagnosis and treatment of BPPV may help to reduce the postconcussion syndrome”. According to Gordon et al. [5], head trauma is the cause of BPPV in about 15% of all cases. The authors reported on 12 cases of traumatic BPPV seen in their clinic and 8 cases diagnosed from 75 consecutive head trauma patients seen in an emergency room. The clinical diagnosis of BPPV was determined using the Hallpike-Dix test, and all patients were treated using the CRM. 40% of all patients reported complete resolution of symptoms after a single treatment session and the remaining patients required several treatments to achieve resolution of symptoms. The authors stated that “traumatic BPPV is probably under-recognized or misdiagnosed in clinical practice” and that the “Hallpike-Dix test is mandatory in all patients with dizziness and vertigo following head trauma”. In another study, Gordon et al. [9] reviewed the clinical records of 247 consecutive patients with BPPV diagnosed using the Hallpike-Dix test and treated with the CRM. The group was subdivided into traumatic BPPV (t-BPPV) and idiopathic BPPV (i-BPPV) groups. Of the 247 patients, 21 (8.5%) fulfilled the diagnostic criteria for t-BPPV. The authors found that 67% of patients with t-BPPV required repeated CRMs for complete resolution in comparison to 14% of patients with i-BPPV (*P* < .001). The authors also discovered that 57% of t-BPPV patients had recurrent attacks compared to 19% of i-BPPV patients who had recurrent attacks (*P* < .004). Motin et al. [6] reported that 13.3% of 150 consecutive acute TBI patients evaluated in a prospective study complained of vertigo. BPPV was confirmed using the Hallpike-Dix test in 50% of those patients and successfully managed using the CRM in all confirmed BPPV patients. The authors concluded that “about half of the patients with severe TBI who complain about positional vertigo suffer from BPPV. These patients can be efficiently treated by physical maneuvers improving the rehabilitation outcome”. Shumway-Cook [10] reported that as many as 65% of TBI patients will eventually experience symptoms of vestibular pathology, including BPPV, at some point during their recovery process, and Shepard et al. [11] state that “BPPV is a common sequel of head injury and is in fact the most common form of posttraumatic vertigo.” The authors go on to say that any TBI patient complaining of vertigo should have a Hallpike-Dix test performed, and if it is positive, a CRM should be used to treat the condition.

## 5. Conclusion

This case report of a patient with chronic recurrent vertigo post-TBI adds to the growing body of evidence that BPPV should be considered during the medical examination of patients suffering from head trauma, especially those reporting vertigo. The clinical performance of both the Hallpike-Dix test and the CRM is relatively simple and requires very little time of the examiner. For a comprehensive review of literature supporting and describing the examination and intervention of BPPV, the reader is referred to the recent work of Fife et al. [2].
